# Introduction

**DOI:** 10.1007/978-3-030-52391-6_1

**Published:** 2020-10-30

**Authors:** Hans Peter Beck, Panagiotis Charitos

**Affiliations:** 1 Albert Einstein Center for Fundamental Physics, Laboratory of High Energy Physics, University of Bern, Bern, Switzerland; 2 Department of Experimental Physics, CERN, Geneva, Switzerland; 4 Albert Einstein Center for Fundamental Physics, Laboratory of High Energy Physics, University of Bern, Bern, Switzerland; 5 Department of Experimental Physics, CERN, Geneva, Switzerland; 3 Hamburg, Hamburg Germany

## Abstract

The present volume collects the proceedings of the workshop “The Economics of Science” that was held in June 2019 in Brussels in the framework of the FCC Week with the support of the H2020 EuroCirCol and EASITrain projects and the Belgium Charter of the LSE Alumni Association. The goal of the meeting was threefold: First to explore the role of public investments in research infrastructures and Big Science projects for economic development, review ways to access their financial impact beyond their core scientific mission and thirdly create a forum for exchanging best practices that can maximize the impact of such projects. The collected essays focus on Big Science Organizations that participated in the workshop while we should clarify to readers that by “Science” we mainly refer to curiosity-driven research. However, we hope that some of the ideas and tools discussed by the participants of the workshop can find applications in many ways.

The present volume collects the proceedings of the workshop “The Economics of Science” that was held in June 2019 in Brussels in the framework of the Future Circular Collider (FCC) Week with the support of the H2020 EuroCirCol and EASITrain projects and the Belgium Charter of the LSE Alumni Association. The goal of the meeting was threefold: First to explore the role of public investments in research infrastructures and Big Science projects for economic development, review ways to access their financial impact beyond their core scientific mission and thirdly create a forum for exchanging best practices that can maximize the impact of such projects. The collected essays focus on Big Science Organizations that participated in the workshop while we should clarify to readers that by “Science” we mainly refer to curiosity-driven research. However, we hope that some of the ideas and tools discussed by the participants of the workshop can find applications in many ways.

The economic and social benefits of Research & Innovation don’t happen by magic; they often have to start with curiosity-driven research, not directed to applications but to explore the nature of our universe and our place in it. What is much less well known is the wider impact this has on technology and our daily lives; fundamental, exploratory science that poses high-risks but also delivers surprising results in tackling some of the most pressing societal problems and unlocking new markets potential. A mere 150 years ago the candle was the main source of artificial light. By now, lighting has been developed to a very sophisticated degree. In Oren Harari’s famous quote: “The electric light did not come from the continuous improvement of candles”. No amount of research on the candle would have given us the electric light bulb which was only made possible through basic science that unveiled the nature of electricity and gave birth to numerous applications. Another example is the phonograph that Edison invented in 1877 based on making bumps in a metal surface and turning them into sound by running a mechanical “finger” along them. Despite years of improvements in the material, development of better bearing and support structures, the real revolution in our listening experience came with the development of MP3 technology. It took a courageous step followed by a long development process to store sound in a digital format thus revolutionizing the quality and volume of sound we can store today. **In conclusion, only focusing on small step improvements that seemingly will lead to the next iteration ready to market, big opportunities will be missed that for short or long will turn out detrimental in any business model, if not in parallel basic and fundamental research are maintained at a healthy level and opportunities that open up from it are embarked on.**

The unprecedented pace of scientific discoveries during the 18th and 19th century that also led to the Industrial Revolution went hand in hand with the development of economics as a separate discipline; developing its own tools and methodology and advancing taking into account the progress of other fields from psychology and sociology to mathematics and computing. However it is our belief that today the pendulum is swinging back and this has been one of the motivations for preparing this volume. The contributions in this publication demonstrate, economists and scientists are coming closer together to realise the strong links between basic research and its societal impact. Today, research facilities, academic institutes, private industry and funding agencies embrace increased multi- and transdisciplinary research to tackle the world’s most challenging problems.

A half-century ago, Gordon Moore wrote a paper in which he projected that progress in the density and speed of silicon chips would increase exponentially. In his paper, Moore envisioned how this would enable technologies ranging from the personal computer, to the smartphone, to the self-driving car.^1^ His prediction became known as Moore’s Law, and it has held remarkably true for 50 years. At the celebration of the fiftieth anniversary of his seminal paper back in 2015, Moore talked about the impact of his insight on modern technology and the crucial role of basic scientific research for realizing it. In his own words: “That’s really where these ideas get started. They take a long time to germinate, but eventually they lead to some marvelous advances. Certainly, our whole industry came out of some of the early understanding of the quantum mechanics of some of the materials. I look at what’s happening in the biological area, which is the result of looking more detailed at the way life works, looking at the structure of the genes and one thing and another. These are all practical applications that are coming out of some very fundamental research.”

The technological progress we have enjoyed over the last century was enabled by the combination of science education and investments in fundamental research. While the opportunities for discovery have never been greater, commitment to and funding for basic science seems to be put under question. It is often seen as absorbing sources from rather more target-oriented research to address major issues that affect our everyday lives such as climate change, infectious diseases, cancer therapies, natural hazards. However this view neglects that although basic science might not offer clear-cut ways to immediately solve problems, it is the bedrock for future fixes. However, these are not “either/or” options but rather based on a “both/and” framework that better describes the positive symbiosis between different fields.

This is why, in our view, it is urgent to foster the dialogue between different actors including researchers, funding agencies, policy makers, economists and innovators among others. We need to learn from previous lessons, exchange current best practises and develop synergies across big scientific organizations that act as hubs for excellent science and catalyzers of global collaboration.

Basic research occurs at a distance from the market, which makes it unappealing as in our society we are more and more used to acting in short- to medium-term timeframes. Once a new idea becomes clear to be useful it still needs a lot of energy and dedication to turn into reality and be used to serve society. The history of science offers ample examples on how the benefits of basic research can take up to decades before translating into innovations and generating a positive return for society. Furthermore, science, and particularly basic science, is also inherently unpredictable about its results, bearing risks and calling for a broader vision that drives scientific progress. Failure is common but success in understanding our world is unmeasurable. How could one quantify the value of discovering X-rays, the study of synchrotron radiation and the fundamental laws of optics about diffraction that played a crucial role from the first moment in uncovering the structure of DNA and the development of new generations of medical therapies? In addition, one should not undermine the value of often unexpected discoveries, the so-called “unknown-unknowns”, like the discovery of the Lamb shift paved the way to Quantum Electro Dynamics (QED) or the surprising observation of spin by Uhlenbeck and Goudsmit in fundamental particles that paved the way for understanding material properties and are crucial when developing new materials with distinct wanted features. Basic research on spin made MRI imaging possible, now widely used in medical diagnostics.

The knowledge generated through curiosity-driven research often disperses into the wider economy as a shared public good thus making it harder to track and quantify the generated revenue. For example in the recent fight against COVID-19 how could one quantify the role of understanding the behavior of complex biological molecules and fundamental research in biology or the role of particle accelerators and electron free lasers in understanding the structures of various aspects of the CoV-2 spike protein through crystallography and electron cryo-microscopy (cryo-EM) and getting valuable information for the development of treatment therapies.

The above mentioned reasons reflect to a certain exchange the challenge of mapping the wider economic impact of curiosity-driven science. Significant efforts in the last decades already shed light to some of the issues tight to this relation. Perhaps at this point we should pause for a moment to reflect on the value that curiosity driven research carries by itself. It is par excellence the field that brings together creative minds to collaborate on curiosity driven research; encouraged to take risks but also be prepared to fail. This is not to undermine the value of more applied-oriented research that transforms these ideas into applications that can benefit society at large. In fact basic and applied research become more intertwined as we are entering the realm of Big Science calling for a broader view. On this note we would like to repeat that most of the essays collected in this volume don’t discuss the direct scientific output of this research - since this is and should remain risk-free following the scientific method and judged by the different scientific communities—but rather trace additional impacts generated through the investment in curiosity-driven scientific efforts.

The contributions in this volume present work done by various research infrastructures and big scientific projects including the European Organization for Nuclear Research (CERN), the European Space Agency (ESA), the European Spallation Source (ESS) and the proposed Square Kilometer Array (SKA). A number of them summarize how these big scientific organizations try to measure the impact that they generate for the society and economy beyond the core scientific questions that these large-scale infrastructures try to answer.

Simon Berry (SKA’s Director of Corporate Strategy) discusses the SKA’s approach in creating a sustainable research infrastructure, well-embedded in local networks and scientific communities while also attracting users from all over the world and generating societal benefits. John Womersley (Director General of the European Spallation Source) outlines some of the key challenges in building sustainable support for any science megaproject, using the European Spallation Source ESS as an example. Thierry Lagrance (CERN, Head of Industry, Procurement and Knowledge Transfer) reflects on CERN’s approach in accelerating and optimising the generation of socio-economic benefits. Charlotte Mathieu (ESA’s Head of the Industrial Policy and Economic Analysis Section) presented the existing framework for assessing the impact of ESA programmes and key lessons derived from past consultations. Finally, Philip Amison (Head of Corporate Strategy and Impact Science at STFC/UKRI) reviews the key findings of an evaluation of the benefits that the UK has derived from CERN based on a 2018 commissioned by STFC and performed by Technopolis. The report captures and measures the range of scientific, economic and social impacts emerging over the past decade, considering both direct UK involvement as well as the wider impact that CERN has on the UK.

Moreover, in this volume, economists and policy makers present the meaning of sustainability in the context of large-scale research infrastructures and some of the existing tools that can inform empirical studies of RI’s socio-economic impact. Margarida Ribeiro (European Commission, Directorate for Research & Innovation) presents the complex and often multi-level sustainability challenges for Research Infrastructures and the key ingredients of a coordinated plan for action among European RIs. Alasdair Reid (Policy Director, European Future Innovation System—EFIS Centre) in his essay offers an overview of the main outcomes of the H2020 RI-PATHS project that aims to provide policy makers, funders and RI managers the tools to assess RI impact on the economy and their contribution to resolving societal challenges. Andrea Bastianin (Ass. Professor, University of Milan—Bicocca) summarizes the results of a social Cost–Benefit Analysis (CBA) of the High Luminosity upgrade of the Large Hadron Collider (HL-LHC) and the merits of this method when applied on Research Infrastructures. His talk followed the presentation of the merits and basic ingredients of a CBA method by Massimo Florio (Professor, University of Milan) arguing that CERN and more generally Big Science Centres (BSCs) are ideal testing grounds for theoretical and empirical economic models while demonstrating the positive net impact that the LHC/HL-LHC has for society. Moreover, Silvia Vignetti (Director, CSIL) in her contribution suggests that to inform strategic planning, the data collection for impact assessment should not be episodic and motivated by external requests from stakeholders and funding agencies but rather a well-integrated activity occurring throughout the lifetime of the infrastructure.

The socio-economic impact of research infrastructures extends over longer time and spatial scales. In her essay, Linn Kretzschman (MSCA ESR, University of Vienna) presented some of the ongoing research on how society can benefit from a new research infrastructure during the design, construction and operation phase. Her work in the Institute of Entrepreneurship and Innovation at Vienna University of Economics (WUW), supported by the H2020 EASItrain project, has identified innovative application fields, outside particle physics, for the required superconducting magnets for a next-generation collider. To identify new market opportunities for superconducting magnets and its manufacturing steps, the team analyzed the full manufacturing value chain with regard to their importance and identified numerous opportunities that were further scrutinized for their research potential. Riccardo Crescenzi (Professor, London School of Economics) discussed how RIs potential for innovation increases when coupled with complementary skills and conditions are available locally to support knowledge generation and absorption. Investments in R&D can enhance regional innovation only when coupled with a supportive endowment of Human Capital. Finally, Maria L. Loureiro (Professor U. Santiago de Compostela, Spain) and Maria Allό (U. Santiago de Compostela, Spain) invite us to rethink the concept of value, how it is defined and offer a more inclusive approach that is appropriate when dealing with global public goods like those created by Big Scientific Infrastructures.

The last part of the volume brings together three essays focusing on the question “Who benefits from such large public investments in science” that informed a public discussion moderated by Mrs. Anjana Ahuja during the last session of the workshop. During the session Massimo Florio (Professor, University of Milan) introduced the question of how Big Science contributes to social justice and can contribute in tackling current inequalities. Two fundamental questions are addressed: What is the economic impact of curiosity-driven research? What are the implications for social justice of the interplay between—on one side—government funded science and—on the other side—R&D supported by business? Arguing for the need to include this dimension on top of discussions about the size of public funding and the socio-economic impact they create. Michela Massimi (Professor, University of Edinburgh) offers her remarks on the importance of fundamental research for society and how it contributes to the human cultural flourishing while arguing that philosophers of science should contribute more and more in the decade to come in this ongoing dialogue and engagement with physicists. Finally, Carsten Welsch (Professor, Head of Physics Department University of Liverpool/Cockcroft Institute) argues that fundamental science informs many aspects of our daily lives and should not be considered as a distant activity and demonstrates that through an extensive discussion of the development and applications that particle accelerators have with more than 50,000 particle accelerators used in industry, for medical treatment and for research.

Closing this editorial we would like to refer to the special impact that research infrastructures for curiosity-driven research have for training the next generation of science. Acting as knowledge and innovation hubs they offer an international and competitive environment, characterized by creativity, collaboration and resilience is key for succeeding in their transition from curiosity-driven research to different sectors where they often contribute in unprecedented ways. The next scientific revolution will be driven by scientists who have a multidisciplinary view of science, the opportunity to take risks, the infrastructure to work, and the freedom to think.

We hope that the publication of these proceedings will inform the greater debate on the value of public and private funding for research infrastructures and inform discussions on the broader value that public investment in curiosity-driven science and research infrastructures for a transition to more resilient and redistributive model of economy in line with the big societal challenges lying ahead in the twenty-first century.

## Footnotes

1 In Moore’s words: “The future of integrated electronics is the future of electronics itself. The advantages of integration will bring about a proliferation of electronics, pushing this science into many new areas. Integrated circuits will lead to such wonders as home computers—or at least terminals connected to a central computer—automatic controls for automobiles, and personal portable communications equipment.” Ref: https://newsroom.intel.com/wp-content/uploads/sites/11/2018/05/moores-law-electronics.pdf

